# Behavioral Intervention Components Associated With Cost-effectiveness: A Comparison of Six Domains

**DOI:** 10.1093/abm/kaab036

**Published:** 2021-06-11

**Authors:** E Beard, F Lorencatto, B Gardner, S Michie, L Owen, L Shahab

**Affiliations:** 1 Research Department of Behavioural Science and Health, University College London, London WC1E 6BT, UK; 2 Department of Clinical, Educational and Health Psychology, Centre for Behaviour Change, University College London, London, UK; 3 Department of Psychology, Institute of Psychiatry, Psychology and Neuroscience, King’s College London, London, UK; 4 National Institute for Health and Care Excellence, NICE, UK

**Keywords:** BCT, Smoking, Diet, Exercise, Sexual health, Alcohol

## Abstract

**Background:**

To help implement behavior change interventions (BCIs) it is important to be able to characterize their key components and determine their effectiveness.

**Purpose:**

This study assessed and compared the components of BCIs in terms of intervention functions identified using the Behaviour Change Wheel Framework (BCW) and in terms of their specific behavior change techniques (BCTs) identified using the BCT TaxonomyV1, across six behavioral domains and the association of these with cost-effectiveness.

**Methods:**

BCIs in 251 studies targeting smoking, diet, exercise, sexual health, alcohol and multiple health behaviors, were specified in terms of their intervention functions and their BCTs, grouped into 16 categories. Associations with cost-effectiveness measured in terms of incremental cost-effectiveness ratio (ICER) upper and lower estimates were determined using regression analysis.

**Results:**

The most prevalent functions were increasing knowledge through education (72.1%) and imparting skills through training (74.9%). The most prevalent BCT groupings were shaping knowledge (86.5%), changing behavioral antecedents (53.0%), supporting self-regulation (47.7%), and providing social support (44.6%). Intervention functions associated with better cost-effectiveness were those based on training (*β*_low_ = −15044.3; *p* = .002), persuasion (*β*_low_ = −19384.9; *p* = .001; *β*_upp_ = −25947.6; *p* < .001) and restriction (*β*_upp_ = −32286.1; *p* = .019), and with lower cost-effectiveness were those based on environmental restructuring (*β* = 15023.9_low_; *p* = .033). BCT groupings associated with better cost-effectiveness were goals and planning (*β*_low_ = −8537.3; *p* = .019 and *β*_upp_ = −12416.9; *p* = .037) and comparison of behavior (*β*_low_ = −13561.9, *p* = .047 and *β*_upp_ = −30650.2; *p* = .006). Those associated with lower cost-effectiveness were natural consequences (*β*_low_ = 7729.4; *p* = .033) and reward and threat (*β*_low_ = 20106.7; *p* = .004).

**Conclusions:**

BCIs that focused on training, persuasion and restriction may be more cost-effective, as may those that encourage goal setting and comparison of behaviors with others.

## Teaser

Behaviour change interventions that focus on training, persuasion and restriction may be more cost-effective, as may those that encourage goal setting and comparison of behaviors with others

## Introduction

Physical inactivity, smoking, excessive alcohol consumption, unprotected sex, and poor diet cost the National Health Service (NHS) in England more than £14 billion per year [1–4] and also adversely affect the local economy [5–7]. Although developing interventions to change behavior (BCIs) is a key objective of public health there is a significant challenge of translating such interventions into routine practice [8]. Many factors contribute to this problem including poor specification of the key components of BCIs [9]. Thus this study aims to provide an evidence synthesis of the key components of BCIs across six domains (smoking, diet, physical activity, alcohol, sexual health, and multiple behaviors) and the association of these with cost-effectiveness using a reliable theory-based coding system [10, 11]. Consideration of cost-effective and not just effective interventions is important as it will aid evidence-based practice and the application of BCIs in the public domain. Part of the failure to implement interventions in the real world results not only from the ability to duplicate the components of the original intervention but also the availability of key resources. Identifying the key components of cost-effective interventions will help decision makers maximize the public’s health with the allocated resources.

BCIs are “coordinated sets of activities designed to change specified behavior patterns,” for example, to help people stop smoking or to increase their exercise levels [10]. BCIs can be characterized in terms of both “content” (active ingredients of the intervention) and “delivery” (manner in which the content is applied, for example, level of intensity of the intervention and setting). Although the complexity of BCIs means that it is not possible to capture every aspect of the content, it is possible to record some key features using coding systems that can be used with an acceptable degree of reliability [11]. One of these coding systems is known as the Behaviour Change Wheel (BCW) [10, 12]. The BCW is a behavioral system, the hub of which specifies that for behavior change to occur one needs three conditions: capability, opportunity and motivation (COM-B). Around this hub, nine intervention functions are positioned which capture ways in which an intervention can change behavior: education, persuasion, incentivization, coercion, training, restriction, environmental restructuring, modeling, and enablement. These intervention functions can then be implemented in an intervention using one of 93 proposed behavior change techniques (BCTs) [11].

Examples of studies that have attempted to identify intervention features associated with effectiveness can be seen across behavioral domains. Diet interventions using the BCTs barrier identification/problem solving, plan social support/social change, goal setting (outcome), use of follow-up prompts, and provide feedback on performance have been associated with greater fruit and vegetable consumption compared with studies not using these BCTs [13]. Physical activity interventions using feedback have been deemed more effective than those not using this, while interventions providing information on where and when to perform the behavior and information on consequences of behavior to the individual appear to be less effective than interventions not using these [14]. Several BCTs have been identified in effective smoking cessation interventions aimed at pregnant smokers including facilitate goal setting, advise on social support, and action planning [15, 16]. It is important to extend these findings to include the association with cost-effectiveness, given that implementing recommendations for providing interventions depends not only on the potential benefits but also on the cost of the intervention under consideration. Although intervention functions and BCTs present in cost-effective interventions have been identified before, with the most prevalent being education and shaping knowledge respectively [17], no study to our knowledge has considered both cost-effective and ineffective interventions and the features commonly associated with each of these.

The current study therefore aimed to:

1. Characterize BCIs according to their intervention functions [10, 12] and BCTs [11].
2. Compare the intervention functions and BCTs used to address smoking, diet, physical activity, alcohol, sexual health, and multiple health behaviors.
3. Identify associations between intervention characteristics and cost-effectiveness (using a threshold of £20,000–£30,000 per quality-adjusted life years (QALY))

## Methods

### Stage 1: Identification and Retrieval of Source Material

The search strategy was conducted by Bazian Ltd for the National Institute for Health and Care Excellence (formally the National Institute for Clinical Excellence) in the databases ECONLIT, NHS EED, and HEED for papers/reviews published between January 2003 and September 2012. Studies were included if they: (a) covered interventions aimed at behavior change in relation to at least one of the following: alcohol, diet, physical activity, sexual behavior, smoking, or multiple behaviors, (b) had conducted an economic analysis, and (c) were randomized controlled trials or systematic reviews published in English. In cases where insufficient detail was provided on intervention content in systematic reviews/meta-analyses, such reviews were excluded as limited resources did not allow retrieval of all primary data and the limited information provided would have biased results. Studies were excluded if they focused on people younger than 16 years and national policy, fiscal and legislative measures.

**Fig. 1.**
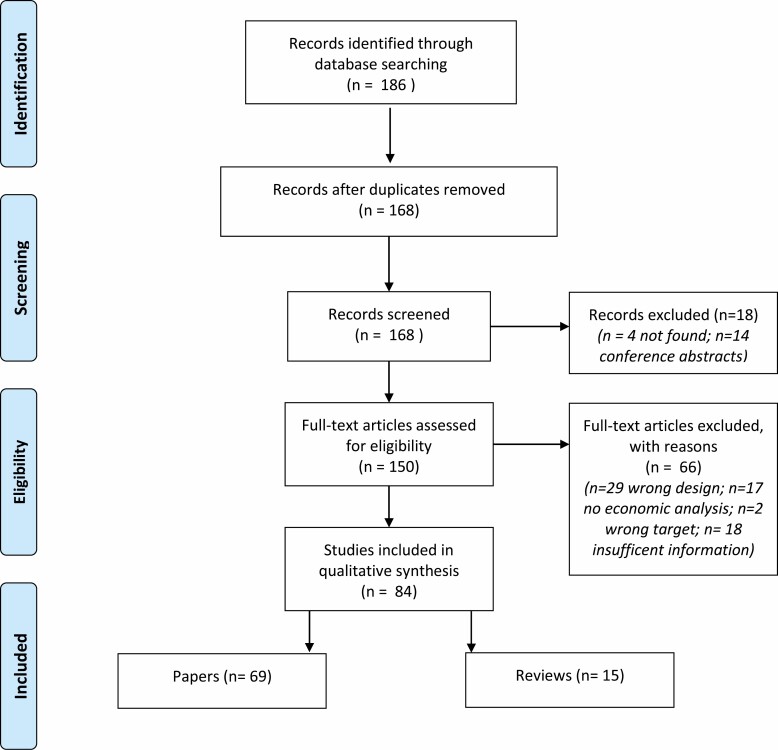
PRISMA flow diagram of included and excluded studies.

Sixty-nine papers and 15 reviews were identified (see Fig. 1), which covered 251 eligible interventions (see Appendix 1 for further details). The majority of these reported cost-effectiveness (CEA) (*n* = 65) or a cost-utility (CUA) (*n* = 102) economic analysis. Both CEA and CUA focus on the cost per unit of health gained of one compared with another intervention, yielding an incremental cost-effectiveness ratio (ICER). While CEA express this in cost per unadjusted health gain (e.g., measures the benefit using single unidimensional outcomes, for example, life-years (LY) saved), CUA is a special form of CEA which also adjusts health benefits for quality of life (e.g., QALY which captures both duration and quality of life). Where necessary estimates were converted into GBP at the time of the original analysis or, when this information was not available, at the time of the paper publication.

As some of the economic assessments carried out sensitivity analyses, varying cost-effectiveness estimates based on several factors such as user characteristics, and time horizons were calculated, and so, both lower (most optimistic) and upper (most pessimistic) limits of cost-effectiveness estimates were recorded. In cases where no sensitivity analysis was carried out, the single estimate was included as both the lower and upper limits. Cost-effectiveness was determined according to the National Institute for Health and Care Excellence (NICE) guidelines. NICE have adopted a CUA-based cost-effectiveness threshold of £20,000–£30,000 per QALY above which interventions are unlikely to be recommended [18].

The remaining 84 interventions used either cost–benefit analysis (CBA) or cost-consequence analysis (CCA) which yield data not expressed in (adjusted) life-years saved. CBA expresses all direct and indirect costs and benefits in a common unit, in monetary terms, and enables calculation of net benefit (unit difference of benefits minus costs). CCA is a special form of CBA which does not attempt to express all costs and benefits in the same unit of measurement and therefore does not aggregate findings into a single indicator.

### Stage 2: Characterization of Interventions

The content of interventions was characterized using two methods. The first identified their intervention functions as defined in the Behaviour Change Wheel (BCW) framework [12] (see Electronic Supplementary Table 1). Interventions were also coded using a taxonomy of 93 BCTs (BCT Taxonomy v1 [11]), with 16 groupings. Following Michie et al.’s [11] guidelines, BCTs were coded only where coders believed that there was unequivocal evidence of their inclusion in a given intervention. All papers were coded by EB and a subset of 28 papers (29.2%) was coded in batches by a second coder (FL) with disagreements resolved through discussion after each batch. Agreement was 99.2%, with a mean Cohen’s kappa of .89, indicating good inter-rater reliability [19].

Interventions were also categorized in terms of a range of factors relating to their context and delivery: intervention level (e.g., individual vs. population), delivery agent type (e.g. health care professional vs. physicians), and intensity (e.g., high vs. medium vs. low e.g. minimal contact, some contact and multiple contacts) [20–22] (see Electronic Supplementary Table 2).

### Analysis

All data were extracted into a data extraction form and then transferred into IBM SPSS. Interventions were assigned to one of six categories: alcohol, diet, smoking, physical activity, sexual-health interventions, and interventions targeting multiple health behaviors. Differences according to intervention characteristics were analyzed using *t*-tests or one-way ANOVAs and *χ*^2^ or Fisher Exact tests for continuous and categorical variables, respectively. The Tukey correction was applied in post hoc analyses.

To meaningfully interpret data and maintain consistency with NICE guidelines, only the 102 interventions where cost-effectiveness status was based on analyses expressed in cost per DALY/QALY gained were included in the primary analysis comparing BCTs of interventions appraised as cost-effective and cost-ineffective according to NICE threshold. Sixty-two percent (*n* = 63) used a single CUA estimate and 38% (*n* = 39) provided multiple upper and lower CUA estimates. Data were analyzed using linear regression models with the estimates entered as a continuous dependent variable.

As a sensitivity analysis, we included all studies with a binary outcome (*n* = 251) of cost-effective versus cost-ineffective. For this variable, cost-effectiveness was based on meeting at least one out of five cost-effectiveness conditions. These conditions were: being below the £20,000 threshold for the upper limit; below £20,000 for the lower limit; below £30,000 for the upper limit; below £30,000 for the lower limit or the original authors’ appraisal that the intervention was cost-effective. Thus if either the lower or upper estimates or both were less than the specified threshold the intervention was deemed cost-effective. Data were analyzed using logistic regression models with the estimate as a binary variable of cost-effective versus not cost-effective.

Unadjusted and adjusted regression models are reported, with variables selected using forward stepwise selection based on the Likelihood Ratio. Analyses were not conducted for individual health behaviors given the small sample size and lack of power.

## Results

More than a third of the 251 identified interventions were smoking cessation interventions (*n* = 92). The next most prevalent category was multiple health-related behavior interventions (*n* = 48) and interventions to improve sexual health (*n* = 44). Thirty-nine interventions considered diet and 28 interventions focused on physical activity, while only 8 alcohol interventions were identified. There was a fairly equal split between different intervention categories with the exception of mass media interventions which featured only five times. Control conditions mostly consisted of usual care, and fewer than 10% of interventions studies used a matched control condition (see Table 1).

**Table 1.** Intervention characteristics by health behavior

	All (*n* = 251)	Smoking (*n* = 92)	Diet (*n* = 39)	Physical activity (*n* = 20)	Alcohol (*n* = 8)	Sexual health (*n* = 44)	Multiple targets (*n* = 48)	p*
Category		a	b	b, c	b, c	c	b, c	
Med	14.7 (37)	29.3 (27)	20.5 (8)	0.0 (0)	0.0 (0)	2.3 (1)	2.1 (1)	
BI	22.3 (56)	13.0 (12)	30.8 (12)	55.0 (10)	75.0 (6)	15.9 (7)	18.8 (9)	
Med + BI	13.9 (35)	26.1 (24)	2.6 (1)	0.0 (0)	0.0 (0)	18.2 (8)	4.2 (2)	<.001
Comp	34.7 (87)	10.9 (10)	38.5 (15)	45.0 (9)	25.0 (2)	43.2 (19)	66.7 (32)	
Med + Comp	12.4 (31)	20.7 (19)	2.6 (1)	0.0 (0)	0.0 (0)	15.9 (7)	8.3 (4)	
Mass media	2.0 (5)	0.0 (0)	5.1 (2)	5.0 (1)	0.0 (0)	4.5 (2)	0.0 (0)	
Control condition		a	a, b	a, b	a, b	b	a, b	
Nothing/UC	66.9 (168)	55.4 (51)	61.5 (24)	75.0 (15)	50.0 (4)	88.6 (39)	72.9 (35)	
Lower impact	25.1 (63)	34.8 (32)	33.3 (13)	20.0 (4)	37.5 (3)	6.8 (3)	16.7 (8)	<.001
Matched impact	8.0 (20)	9.8 (9)	5.1 (2)	5.0 (1)	12.5 (1)	4.5 (2)	10.4 (5)	
Intervention intensity		a	b	a, b	a, b, c	a	c	
Unclear	4.8 (12)	0.0 (0)	12.8 (5)	5.0 (1)	0.0 (0)	2.3 (1)	10.4 (5)	
Low	38.2 (96)	57.6 (53)	43.6 (17)	40.0 (8)	25.0 (2)	29.5 (13)	6.2 (3)	*<.001*
Medium	24.3 (61)	20.7 (19)	10.3 (4)	30.0 (6)	25.0 (2)	40.9 (18)	25.0 (12)	
High	32.7 (82)	21.7 (20)	33.3 (13)	25.0 (5)	50.0 (4)	27.3 (12)	58.3 (28)	
Setting		a	b, c	c	a, b	b, c	b, c	
Primary care	65.3 (164)	85.9 (79)	53.8 (21)	40.0 (8)	75.0 (6)	65.9 (29)	43.1 (21)	
Secondary care	6.4 (16)	2.2 (2)	15.4 (6)	0.0 (0)	25.0(2)	0.0 (0)	12.5 (6)	
Community	17.1 (43)	7.6 (7)	15.4 (6)	35.0 (7)	0.0 (0)	20.5 (9)	29.2 (14)	*<.001*
Workplace	1.6 (4)	3.3 (3)	2.6 (1)	0.0 (0)	0.0 (0)	0.0 (0)	0.0 (0)	
Unclear/other≠	9.6 (24)	1.1 (1)	12.8 (5)	25.0 (5)	0.0 (0)	13.6 (6)	14.6 (7)	
Delivery mode		a	a, b	b	a, b	a, b	a, b	
Physician	9.2 (23)	15.4 (14)	17.9 (7)	0.0 (0)	0.0 (0)	2.3 (1)	2.1 (1)	
HP	66.4 (166)	65.9 (60)	43.6 (17)	65.0 (13)	100.0 (15)	77.3 (34)	70.8 (34)	
Media	4.0 (10)	0.0 (0)	7.7 (3)	15.0 (3)	0.0 (0)	4.5 (2)	4.2 (2)	*.015*
Mix	4.4 (11)	4.4 (4)	7.7 (3)	5.0 (1)	0.0 (0)	2.3 (1)	4.2 (2)	
Unclear/other±	16.0 (40)	14.3 (13)	23.1 (9)	15.0 (3)	0.0 (0)	13.6 (6)	18.8 (9)	
Target level								
Individual	84.1 (211)	94.6 (87)	74.4 (29)	85.0 (17)	62.5 (5)	79.5 (35)	79.2 (38)	
Groups	8.4 (21)	3.3 (3)	10.3 (4)	10.0 (2)	37.5 (3)	6.8 (3)	12.5 (6)	*.037*
Population	2.4 (6)	0.0 (0)	5.1 (2)	5.0 (1)	0.0 (0)	4.5 (2)	2.1 (1)	
Mix	5.2 (13)	2.2 (2)	10.3 (4)	0.0 (0)	0.0 (0)	9.1 (4)	6.2 (3)	
Population		a	b,c	b,c	b,c	b	c	
General	49.0 (123)	14.1 (13)	64.1 (25	55.0 (11)	87.5 (7)	56.8 (25)	87.5 (42)	*<.001*
Vulnerable*	51.0 (128)	85.9 (79)	35.9 (14)	45.0 (9)	12.5 (1)	43.2 (19)	12.5 (6)	
Supporting material								
None	70.5 (177)	69.6 (64)	76.9 (30)	55.0 (11)	87.5 (7)	79.5 (35)	62.5 (30)	
Self-help	9.2 (23)	14.1 (13)	7.7 (3)	15.0 (3)	12.5 (1)	2.3 (1)	4.2 (2)	.178
Electronic	10.4 (26)	8.7 (8)	5.1 (2)	15.0 (3)	0.0 (0)	9.1 (4)	18.8 (9)	
Mix	10.0 (25)	7.6 (7)	10.3 (4)	15.0 (3)	0.0 (0)	9.1 (4)	14.6 (7)	
Pharmacological support	43.8 (110)	78.3 (72)a	33.3 (13)b	0 (0)c	0.0 (0)b,c	36.4 (16)b	18.8(9)b,c	*<.001*
Social marketing	4.0 (10)	0 (0)	5.1 (2)	15.0 (3)	0 (0)	6.8 (3)	4.2 (2)	*.025*
Incentives	4.4 (11)	4.3 (4)	2.6 (1)	20.0 (4)	0 (0)	2.3 (1)	2.1 (1)	0.116
Cost-effective^	87.6 (220)	93.5 (86)a	84.6 (35)a, b	95.0 (21)a, b	100.0 (8)a, b	88.6 (44)a, b	72.9 (36)b	*0.012*

Note: ^≠^Refers to state/policy level interventions (e.g., changes in legislation/physical infrastructure) or interventions in nonspecific settings (e.g., online/phone interventions).

^±^This refers to delivery by peers, teachers, researchers or the state.

^ Based on CUA (*N* = 102) and non-CUA (*N* = 149) cost-effectiveness studies.

*Vulnerable includes pregnant women, individuals at risk of disease, those from low socio-economic groups and patient populations.

a, b, c: Comparison of interventions targeting different health-related behaviors, different letters indicate significant difference at *p* < .05 (Bonferroni-corrected).

BI, brief intervention; Med, medication; Comp, comprehensive; UC, usual care; HP, healthcare professional (nurse, pharmacist, psychologist etc.).

### Broad Characterization of Interventions

#### Overall

Table 1 shows the broad characteristics of the identified interventions. Over a third of interventions were classified as being of low intensity (i.e., mostly brief or pharmacological interventions), mainly set in primary care and were delivered by health professionals. Interventions most frequently targeted individuals from both general and vulnerable populations (e.g., pregnant women, individuals at risk of disease, those from low socio-economic groups and patient populations). Over four-fifths of interventions were considered cost-effective.

#### By behavioral domain

Smoking cessation interventions compared with others most commonly involved medication. The majority of smoking cessation and alcohol interventions were set in primary care compared with less than half of physical activity interventions. While the majority of smoking cessation and sexual health intervention was aimed at vulnerable populations, other heath behavior interventions tended to target the general population, in particular those aimed at changing multiple behaviors (see Table 1)

### Intervention Functions

#### Overall

The most prevalent functions, identified in three-quarters of interventions, were to increase knowledge and/or understanding through education as well as to impart skills through training (see Table 2). Nearly half of the interventions aimed to increase capability and/or opportunity and a quarter of interventions used persuasion to encourage behavior change. Environmental restructuring by changing physical or social contexts or using incentives to create an expectation of reward was relatively uncommon as were restriction and modeling, while none used coercion.

**Table 2.** Intervention characteristics by health-related behavior

	All (*n* = 251)	Smoking (*n* = 92)	Diet (*n* = 39)	Physical activity (*n* = 20)	Alcohol (*n* = 8)	Sexual health (*n* = 44)	Multiple targets (*n* = 48)	p*
Training	74.9 (188)	78.2 (72)a,c	92.3 (36)c	35.0 (7)b	50.0 (4)a,b,c	56.8 (25)a,b	91.7 (42)c	*<.001*
Education	72.1 (181)	62.0 (57)a	66.7 (26)a,b	65.0 (13)a,b	100.0 (8)a,b	77.3 (34)a,b	89.6 (43)b	*.001*
Enablement	45.8 (115)	72.8 (67)a	30.8 (12)b	10.0 (2)b	0 (0)b	36.4 (16)b	37.5 (18)b	*<.001*
Persuasion	24.7 (62)	33.7 (31)a,c	2.6 (1)b	35.0 (7)a,c	62.5 (5)a	27.3 (12)a,c	12.5 (6)b,c	*<.001*
Environmental restructuring	5.2 (13)	6.5 (6)	7.7 (3)	15.0 (3)	0 (0)	0 (0)	2.1 (1)	.071
Incentivization	4.0 (10)	4.3 (4)	2.6 (1)	10.0 (2)	0 (0)	2.3 (1)	4.2 (2)	.730
Restriction	2.8 (7)	0 (0)a	15.4 (6)b	0 (0)a,b	0 (0)a,b	0 (0)a,b	2.1 (1)a,b	*.001*
Modelling	1.2 (3)	0 (0)	0 (0)	0 (0)	0 (0)	2.3 (1)	4.2 (2)	.273
Coercion	0 (0)	0 (0)	0 (0)	0 (0)	0 (0)	0 (0)	0 (0)	NC

Note: *Significant overall differences are in italics.

a, b, c: Comparison of interventions targeting different health-related behaviors, different letters indicate significant difference at *p* < .05 (Bonferroni-corrected).

NC, cannot be computed.

#### By behavioral domain

Intervention functions differed according to the health-related behavior targeted. The use of training was significantly more prevalent in diet interventions and interventions aimed at changing multiple behaviors than in physical activity and sexual health interventions. The use of education was lowest in smoking cessation interventions compared with multiple behavior interventions. Smoking cessation interventions were much more likely than interventions aimed at changing any other health-related behavior to employ the enablement function (see Table 2). Alcohol and smoking cessations were also more likely than interventions targeting multiple health-related behaviors or diet interventions to employ persuasion, whereas restriction was more prevalent in the diet than in smoking cessation interventions.

### BCT Groupings in Interventions

#### Overall

BCTs were grouped as shown in Table 3. Out of a total of 16 BCT groupings, four (shaping knowledge, antecedents, regulation and social support) were particularly prevalent, evident in about half of the interventions considered. A further five BCT groupings (comparison of outcomes, feedback and monitoring, goals and planning, natural consequences, and self-beliefs) were commonly identified, evident in a fifth to a third of interventions. The remainder were coded in less than 10% of interventions with very few using identity, scheduled consequences and covert learning.

**Table 3.** BCT groupings and total number of BCTs by health-related behavior

	All (*n* = 251)	Smoking (*n* = 92)	Diet (*n* = 39)	Physical activity (*n* = 20)	Alcohol (*n* = 8)	Sexual health (*n* = 44)	Multiple targets (*n* = 48)	p*
% (*n*)								
Shaping knowledge (BCT36–39)	86.5 (217)	92.4 (85)a,c	89.7 (35) a,b,c	55.0 (11)b	50.0 (4)a,b	95.5 (42)c	83.3 (40)a,b,c	*<.001*
Antecedents (BCT30–35)	53.0 (133)	76.1 (70)a	56.4 (22)a,b	35.0 (7)b,c	0 (0)c	43.2 (19)b,c	31.2 (15)b,c	*<.001*
Regulation (BCT4–7)	47.7 (119)	78.3 (72)a	35.9 (14)b,d	0 (0)c	0 (0)b,c	52.3 (23)b	20.8 (10)c,d	*<.001*
Social support (BCT1–3)	44.6 (112)	43.5 (40)	33.3 (13)	50.0 (10)	87.5 (7)	38.6 (17)	52.1 (25)	.062
Comparison of outcomes (BCT74–BCT76)	37.1 (93)	44.6 (41)a,b	20.5 (8)a	25.0 (5)a,b	25.0 (2)a,b	22.7 (10)a	56.3 (27)b	*.001*
Feedback and monitoring (BCT8–14)	27.1 (68)	9.8 (9)a	38.5 (15)b	20.0 (4)a,b	75.0 (6)b	25.0 (11)a,b	47.9 (23)b	*<.001*
Goals and planning (BCT65–73)	26.7 (67)	16.3 (15)a	20.5 (8)a	30.0 (6)a,b	50.0 (4)a,b	20.5 (9)a	52.1 (25)b	*<.001*
Natural consequences (BCT82–87)	22.7 (57)	8.7 (8)a	23.1 (9)a,b	15.0 (3)a,b	12.5 (1)a,b	45.5 (20)b	33.3 (16)b	*<.001*
Self-beliefs (BCT40–43)	22.3 (56)	28.3 (26)a	2.6 (1)b	40.0 (8)a	37.5 (3)a,b	27.3 (12)a	12.5 (6)a,b	*<.001*
Repetition and substitution (BCT23–29)	10.0 (25)	0 (0)a	7.7 (3)a	10.0 (2)a,b	0 (0)a,b	4.5 (2)a	37.5 (18)b	*<.001*
Comparison of behavior (BCT88–90)	9.6 (24)	1.1 (1)a	7.7 (3)a,b	0 (0)a,b	0 (0)a,b	13.6 (6)a,b	29.2 (14)b	*<.001*
Associations (BCT15–22)	9.2 (23)	8.7 (8)	7.7 (3)	15.0 (3)	0 (0)	9.1 (4)	10.4 (5)	.783
Reward and threat (BCT54–64)	6.8 (17)	6.5 (6)	2.6 (1)	20.0 (4)	0 (0)	2.3 (1)	10.4 (5)	.107
Identity (BCT77–81)	0.8(2)	1.1 (1)	0 (0)	0 (0)	0 (0)	0 (0)	2.1 (1)	.768
Scheduled consequences (BCT44–53)	0 (0)	0 (0)	0 (0)	0 (0)	0 (0)	0 (0)	0 (0)	NC
Covert learning (BCT91–93)	0 (0)	0 (0)	0 (0)	0 (0)	0 (0)	0 (0)	0 (0)	NC
Mean (range)								
Number of BCT groupings	4.0 (1-9)	4.2 (1-8)a,b	3.5 (1-6)a	3.2 (1-7)a	3.4 (2-4)a,b	4.0 (2-7)a,b	4.8 (1-9)b	*<.001*

Note: *Significant overall differences are in italics.

a, b, c, d: Comparison of interventions targeting different health-related behaviors, different letters indicate significant difference at *p* < .05 (Bonferroni-corrected).

NC, cannot be computed.

#### By behavioral domain

The prevalence of BCT groupings differed by behavioral domain (see Table 3). Shaping knowledge and the use of antecedents was prevalent in smoking, diet, and sexual health interventions. Regulatory BCTs were particularly prevalent in smoking cessation interventions but not present in physical activity or alcohol interventions. Interventions with multiple behavioral targets often focused on comparison of outcomes and goals and planning, the former being less prevalent in diet and sexual health interventions and the latter in smoking cessation interventions. The use of feedback and monitoring was most prevalent in interventions for diet, alcohol and multiple health behavior targets but relatively rare in smoking cessation interventions. BCTs concerning self-beliefs were particularly uncommon in diet interventions. BCTs that highlighted natural consequences were present in nearly half of sexual health interventions and a third of interventions with multiple behavioral targets but relatively uncommon in other interventions, particularly smoking cessation interventions. Smoking cessation also rarely include BCTs that involved repetition and substitution or which instigated comparison of behavior, particularly when compared with multiple health-related behavior interventions. The only BCT grouping that was equally present across all health-related behavioral interventions was social support.

### BCTs in Interventions

#### Overall

**Fig. 2.**
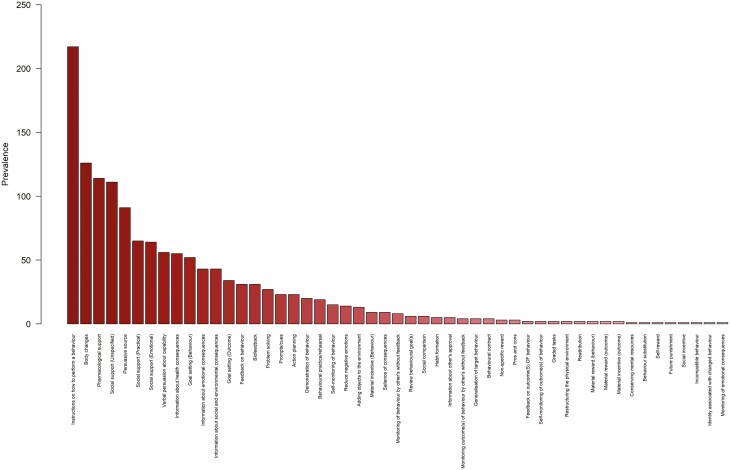
Prevalence of individual BCTs across all interventions. Note: Only BCTs described in at least one interventions are shown.

Out of 93 possible BCTs, the average intervention contained just five BCTs. A total of 51 BCTs were coded for in at least one interventions (see Fig. 2). Instructions on how to perform a behavior (e.g., advise the person how to use nicotine replacement therapy) was by far the most prevalent of BCTs, being reported in nearly nine out of ten interventions. Body changes (e.g., prompt strength training), pharmacological support (e.g., suggest the patient asks the family physician for nicotine replacement therapy) and unspecified social support (e.g., live information about a self-help alcohol group) were also prevalent in nearly half of all interventions analyzed. A third of interventions made use of a persuasive source (e.g., present a speech given by a high status professional to emphasize the importance of a healthy diet) and a quarter included practical or emotional social support (e.g., ask a partner of the patient to exercise with them) or used verbal persuasion (e.g., tell the person they can successfully increase their physical activity)to increase capability.

#### By behavioral domain

There were significant differences in the number of BCTs included in interventions across the behavioral domains (*F*(5, 245) = 4.29, *p* = .001). Interventions with multiple behavior targets included significantly more BCTs (mean = 7.1, median = 6.5, mode = 4), than smoking cessation (mean = 4.9, median = 4, mode = 4; *p* = .001), diet (mean = 4.7, median = 4, mode = 3; *p* = .005) and physical activity (mean = 4.6, median = 4, mode = 4; *p* = 0.042) interventions. There were no differences in the number of BCTs included in sexual health (mean = 5.9, median = 5, mode = 3) or alcohol interventions (mean = 5.8, median = 6, mode = 7).

A total of 30 distinct BCTs were coded at least once in 92 smoking cessation interventions, 29 BCTs in 39 diet interventions and 27 BCTs in 20 physical activity interventions (see Electronic Supplementary Figs. 1–3). The most prevalent BCT (recorded in over 90% of the diet and smoking cessation interventions and over 50% of physical activity interventions) was the inclusion of instructions on how to perform a behavior. The next most prevalent used BCT in diet and smoking cessation interventions was bodily changes, while nonspecific support was the second most prevalent in physical activity interventions. Three-quarters of smoking cessation interventions also discussed providing pharmacological support and just under half involved the inclusion of a persuasive source and provided nonspecific social support. Around a third of diet interventions also provided unspecified social support and pharmacological support and a quarter included practical and emotional social support.

Alcohol interventions included the fewest number of BCTs (*n* = 13) (see Electronic Supplementary Fig. 4). Nearly all alcohol interventions provided unspecified social support and three quarters also offered practical and emotional social support and provided biomarker feedback (e.g., inform the person of their blood pressure reading to improve adoption of health behaviors). Across 44 sexual health interventions, 30 BCTs were coded (see Electronic Supplementary Fig. 5). Most of the interventions included instructions on how to perform a desired behavior and around half also provided pharmacological support or information about health consequences (e.g., present the likelihood of contracting a sexually transmitted infection following unprotected sexual behavior). Finally, 40 BCTs were coded across the 48 interventions targeting multiple behaviors (see Electronic Supplementary Fig. 6). The most common ones were instructions of how to perform a behavior, persuasive source, and social support.

### Cost-effectiveness of the Behavioural Interventions

When looking at the lower but not higher bound estimates (*F*(5, 96) = 0.972, *p* = .439) of cost-effectiveness estimates provided in the 102 cost-utility analyses, significantly more smoking cessation interventions than interventions targeting multiple health-related behaviors were considered cost-effective (*F*(5, 96) = 3.47, *p* = .006) (see Table 4). Similarly, using the £20,000 threshold to define cost-effectiveness, only with the lower estimate (*F*(5, 96) = 2.356, *p* = .046) but not the higher estimate (*F*(5, 96) = 0.961, *p* = .446) was there a significant difference between smoking cessation and multiple health-related behavior interventions (see Table 4). By contrast, when using the £30,000 threshold to define cost-effectiveness, there were no differences between interventions.

**Table 4:** Prevalence of cost-effective interventions as a function of NICE threshold and lower/upper cost-effectiveness estimates

	All (*n* = 102)	Smoking (*n* = 37)	Diet (*n* = 20)	Physical activity (*n* = 3)	Alcohol (*n* = 2)	Sexual health (*n* = 13)	Multiple targets (*n* = 27)	p*
Mean (SEM)								
Lower estimate	£8,025 (1,528)	£2,382a (648)	£6,098a,b (1,265)	£109a,b (109)	£4,858a,b (572)	£11,012a,b (6,360)	£16,864b (4,286)	.006
Higher Estimate	£11,470 (1,898)	£7,593 (2,769)	£11,812 (3,714)	£4,962 (4,962)	£4,858 (572)	£11,763 (6,540)	£17,601 (4,310)	.439
% (N) below £20,000 threshold								
Lower estimate	91.2 (93)	100.0 (37)a	95.0 (19)a,b	100.0 (3)a,b	100.0 (2)a,b	84.6 (11)a,b	77.8 (21)b	.046
Higher Estimate	85.3 (87)	91.9 (34)	85.0 (17)	100.0 (3)	100.0 (2)	84.6 (11)	74.1 (20)	.446
% (N) below £30,000 threshold								
Lower estimate	95.1 (97)	100.0 (37)	100.0 (20)	100.0 (3)	100.0 (2)	92.3 (12)	85.2 (23)	.105
Higher Estimate	90.2 (92)	94.6 (35)	95.0 (19)	100.0 (3)	100.0 (2)	84.6 (11)	81.5 (22)	.473

Note: *Significant overall differences are in italics.

a, b, c: Comparison of interventions targeting different health-related behaviors, different letters indicate significant difference at *p* < .05 (Bonferroni-corrected).

#### Factors associated with cost-effectiveness

In the stepwise adjusted regression model for the continuous limit (see Table 5) interventions using a matched impact control group were associated with less cost-effectiveness than those based on usual care (*β* = 13480.1, *p* = .003), those of high intensity were also associated with less cost-effectiveness than those of low intensity (*β* = 14140.3, *p* = .001). In contrast, those targeting groups (*β* = 16628.1, *p* < .001) or a mixture of groups and individuals (*β* = 19666.4, *p* < .001) were associated with more cost-effectiveness than those targeting individuals only, as were those recruiting non-vulnerable participants (*β* = −10858.3, *p* = .001). Those offering self-help supporting materials were associated with less cost-effectiveness than those not offering any such materials (*β* = 14783.3, *p* = .003). In terms of intervention functions, those based on training (*β* = −15044.3, *p* = .002) and persuasion (*β* = −19384.9, *p* = .001) were associated with more cost-effective, and those based on environmental restructuring were associated with less cost-effectiveness (*β* = 15023.9, *p* = .033). Several BCT groupings were also associated with greater (goals and planning *β* = −8537.3, *p* = .019; comparison of behavior *β* = −13561.9, *p* = .047) or lower cost-effectiveness (natural consequences *β* = 7729.4, *p* = 0.033; reward and threat *β* = 20106.7, *p* = 0.004).

**Table 5.** Factors associated with continuous upper and lower cost-effectiveness estimates (based on CUA studies *N* = 102)

	Lower cost-effectiveness estimate						Upper cost-effectiveness estimate					
	Unadjusted			Adjusted			Unadjusted			Adjusted		
	*Β*	95% CI	*p*	*β*	95% CI	*p*	*β*	95% CI	*p*	*Β*	95% CI	*p*
Category												
Med	Ref						Ref					
BI	6188.8	−3927.8 to 16305.5	0.228				−7676.3	−20302.2 to 4949.7	0.230			
Med + BI	212.7	−11601.0 to 1206.4	0.972				−12642.5	−27386.5 to 2101.5	0.092			
Comp	9831.8	909.7 to 18753.8	0.031*				−1645.7	−127780.7 to 9489.4	0.770			
Med + Comp	1621.7	−9896.6 to 13140.0	0.780				12062.5	−26437.8 to 2312.9	0.099			
Mass media	−2020.8	−21006.3 to 16946.7	0.832				−16103.9	−39787.3 to 77579.6	0.180			
Control condition												
Nothing/UC	Ref			Ref			Ref					
Lower impact	−1924.9	−9366.7 to 6616.8	0.609	3878	−2800.5 to 10556.4	0.251	−183.4	−9556.6 to 9189.8	0.969			
Matched impact	8384.8	−1482.9 to 18252.5	0.095	13480.1	4857.5 to 22102.8	0.003*	5099.5	−77329.3 to 17528.2	0.418			
Intervention intensity												
Low	Ref			Ref			Ref			Ref		
Medium	6100.6	−149.8 to 13951.1	0.126	3866.2	−3430.3 to 11162.7	0.294	1486.6	−8346.7 to 11319.8	0.765	−353.5	−10660.1 to 9953.2	
High	7140.5	−360.3 to 14641.3	0.062	14140.3	6302.8 to 21977.8	0.001*	6405.6	−2989.8 to 15800.9	0.179	13071.2	1686.8 to 24455.6	0.946
Unclear	3397.7	−8018.6 to 14814.0	0.556	998.1	−9118.6 to 11114.8	0.845	−1972.4	−16272.1 to 12327.4	0.785	−16263	−32726.4 to 200.4	0.025*
Setting												.053
Primary care	Ref						Ref					
Secondary care	−1459.3	−14648.3 to 117729.6	0.827				−6838.2	−23280.5 to 9604.1	0.411			
Community	5121.1	−2657.3 to 11729.6	0.827				−*231.6*	−9928.8 to 9465.5	*0.962*			
Workplace	−7306.3	−38420.1 to 23807.4	0.642				−47785.2	−43573.7 to 34003.3	0.807			
Unclear/other	−1647.0	−11734.4 to 8440.5	0.747				−6662.2	−19237.9 to 5913.5	0.296			
Delivery mode												
Physician	Ref						Ref			Ref		
HP	7416.0	−2442.8 to 17274.9	0.139				12519.8	453.1 to 24586.5	0.042*	−6717.8	−23311.1 to 9875.6	.422
Media	7784.8	−7579.0 to 23148.7	0.137				7213.8	−11590 to 26018.4	0.448	−14227.7	−48178.7 to 19723.3	.406
Mix	−967.9	−15581.9 to 13646.0	0.896				4162.0	−13724.8 to 22048.8	0.645	−9358.9	−32811.5 to 14093.7	.429
Unclear/other±	3003.3	−7787.9 to 13794.5	0.582				3012.0	−10195.9 to 16219.9	0.652	−14518.1	−33014.3 to 3978.1	.122
Target level												
Individual	Ref			Ref			Ref			Ref		
Groups	−3031.7	−12236.9 to 6173.4	0.515	−16628.1	−24982.3 to −8274	<0.001*	−4911.9	−16339.6 to 6515.7	0.396	−21653.4	−34161.5 to −9145.3	.001*
Population	−8344.7	−26425.4 to 97746.0	0.362	15176	−13703.3 to 44055.2	0.298	−12199.3	−34577.8 to 10339.2	0.287	49323.7	1980.1 to 96667.4	.041*
Mix	−6701.6	−18830.2 to 5426.9	0.276	−19666.4	−31361.8 to −7971	0.001*	−6405.4	−21462.2 to 8651.5	0.401	−20204.7	−37115 to −3294.3	.020*
Population												
Vulnerable	Ref			Ref						Ref		
General	−97794.3	−15590.2 to −3998.4	0.001*	−10858.3	−16932.3 to −4784.3	0.001*	−11784.0	−19007.2 to −4560.8	0.002*	−24183.7	−33696.4 to −14671	<.001*
Supporting material												
None	Ref			Ref						Ref		
Self-help	−1345.77	−11049.2 to 8357.7	0.784	−4025.6	−13579.3 to 5528.1	0.404	14770.1	−10920.9 to 13861.1	0.814	9361.7	−4894.3 to 23617.7	.195
Electronic	8478.1	−1225.3 to 18181.6	0.086	14783.3	5134.5 to 24432.1	0.003*	6104.4	−6286.6 to 18495.4	0.331	14386.2	441.4 to 28331	.043*
Mix	11602.4	1488.3 to 21716.4	0.025*	9832.3	−1127.2 to 20791.7	0.078	7836.6	−5078.7 to 207751.1	0.231	−1541.1	−21343.3 to 18261.1	0.877
Pharmacological support	−4047.7	−10165.7 to 2070.4	0.192	−14847.2	−31139.4 to 1445	0.073	−221.5	−77885.0 to 7442.1	0.954			
Social marketing	−4460.0	−18545.0 to 9624.9	0.531	−22411.5	−42218.2 to −2604.8	0.027*	−7966.6	−25422.9 to 9489.7	0.367	−31072.9	−58935.4 to −3210.4	.029*
Incentives	−5782.3	−36686.9 to 25122.3	0.711				−9260.7	−47625.9 to 29104.5	0.633			
Intervention functions												
Training	−3078.1	−10728.4 to 4572.2	0.427	−15044.3	−24157.8 to −5930.8	0.002*	−9260.7	−4625.9 to 29104.5	0.633	−6439.9	−18308.2 to 5428.3	.283
Education	6572.1	−130.2 to 13274.3	0.055				933.5	−8596.5 to 10463.5	0.846			
Enablement	−2494.0	−8665.5 to 3677.5	0.425				235.77	−5727.0 to 11198.4	0.523			
Persuasion	−4394.6	−11633.5 to 2844.4	0.231	−19384.9	−30087 to −8682.9	0.001*	1565.9	−6117.3 to 9249.0	0.687	−25947.6	−38411.8 to −13483.4	<.001*
Environmental restructuring	−3626.1	−17621.4 to 10569.3	0.621	15023.9	1272.7 to 28775.1	0.033*	−8241.5	−177148.2 to 655.3	0.069	16475.1	−2072.7 to 35022.8	.081
Incentivization	13003.4	−8821.6 to 34828.4	0.240				−7147.9	−24618.1 to 10322.4	0.419	21868.6	−7845.9 to 51583.1	.147
Restriction	−1834.3	−17527.9 to 13859.3	0.817				9490.2	−17739.6 to 36720.0	0.491	−32286.1	−59066.5 to −5505.8	.019*
Modeling	−1370.3	−32295 to 29554.3	0.930	−18938.3	−45216.1 to 7339.5	0.155	−5419.1	−24885.8 to 14047.5	0.582	−27701.8	−63850.8 to 8447.1	.131
Coercion	NA	NA	NA	NA	NA	NA	−4648.7	−43245.8 to 33548.4	0.803		NA	NA
BCT groupings												
Shaping knowledge	813.5	−8039.8 to 9666.8	0.856	8856.8	−105.6 to 17819.2	0.053	NA	NA	NA			
Antecedents	−2359.9	−8446.6 to 3726.9	0.444				47887.77	−6167.6 to 157745.0	0.388			
Regulation	−2058.4	−8149.5 to 4032.6	0.504	11193.4	−3264.9 to 25651.6	0.127	−105.8	−7687.7 to 7476.1	0.9778			
Social support	2430.4	−3674.3 to 8535.0	0.431				1008.9	−65770.4 to 8588.1	0.792			
Comparison of outcomes	−379.4	−9516.3 to 8757.6	0.935	3907.6	−1759.9 to 9575.1	0.174	−148.5	−7753.8 to 77456.9	0.969	−8906.1	−18760 to 947.9	.076
Feedback and monitoring	3813.2	−3330.2 to 10956.7	0.292				−3450.9	−14788.5 to 77876.6	0.547			
Goals and planning	3812.8	−2973.0 to 10598.77	0.268	−8537.3	−15613.6 to −1461	0.019*	25778.4	−6328.5 to 11485.3	0.567	−12416.9	−24062.3 to −771.5	.037*
Natural consequences	7775.6	1124.2 to 14426.9	0.022*	7729.4	661.6 to 14797.3	0.033*	1307.0	−7169.1 to 9783.1	0.760			
Self-beliefs	−2914.9	−10886.8 to 5057.1	0.470	6848.6	−3182.7 to 16879.9	0.178	6848.0	−1522.6 to 15218.5	0.108			
Repetition and substitution	492.9	−9753.5 to 107739.2	0.924	14037.2	−47.4 to 28121.9	0.051	−6101.2	−15954.2 to 3751.7	0.222	14828.5	−7033.1 to 36690	.181
Comparison of behavior	−3779.4	−9516.3 to 8757.6	0.935	−13561.9	−26950.5 to −173.3	0.047*	−2224.4	−14943.0 to 10494.3	0.729	−30650.2	−52403 to −8897.3	.006*
Associations	3186.1	−14827.3 to 21209.6	0.727				−3450.9	−14778.5 to 7876.6	0.547			
Reward and threat	23339.0	10007.5 to 36670.5	0.001	20106.7	6750.8 to 33462.7	0.004*	−362.5	−22760.9 to 22035.8	0.974			
Identity	11355.7	−19487.9 to 42199.4	0.467				19717.2	2631.6 to 36802.7	0.024*	37685.8	−3784.3 to 79155.9	.074
Scheduled consequences	NA	NA	NA				77877.4	−30500.0 to 46254.7	0.685			
Covert learning	NA	NA	NA				NA	NA	NA			
Number of BCTS	1256.2	2877.6 to 2224.7	0.012*				991.1	−235.3 to 2217.5	0.112	1763.1	−243.3 to 3769.6	.084

Note: * indicates significance, lower and upper cost-effectiveness analyses were based on *n* = 102 studies which provided cost-utility analyses.

NA, not applicable as this category was not coded in any intervention.

In the stepwise adjusted model for the upper limit (see Table 5), interventions of high intensity were associated with less cost effective than those of low intensity (*β* = 13071.2, *p* = .025), as were those aimed at the population compared to individuals (*β* = 49323.7, *p* = .041), while those aimed at groups or a mixture were associated with more cost-effectiveness (*β* = −21652.4, *p* = .001 and *β* = −20204.7, *p* = .020). Interventions recruiting the general population were generally associated with more cost-effectiveness (*β* = −24183.7, *p* < .001) as were those based on social marketing (*β* = −31072.9, *p* = .029). The functions persuasion and restriction were associated with higher cost-effectiveness (*β* = −25947.6, *p* < .001 and *β* = = 32286.1, *p* = 0.019) as were the BCT groupings goals and planning and comparison of behavior (*β* = −12416.9, *p* = .037 and *β* = −30650.2, *p* = .006).

In sensitivity analyses using a binary estimate of cost-effectiveness involving all 251 studies, those studies where the control condition was classified as having a lower impact (being less comprehensive) had higher odds of being cost-effective compared with those with a standard control condition, that is, receiving usual care (OR 5.374, 95% CI 1.200 to 23.674, *p* = 0.026). Additionally, higher intervention intensity was associated with lower cost-effectiveness (OR 0.283, 0.112 to 0.727, *p* = .009) (see Table 6).

**Table 6.** Factors associated with binary cost effective versus cost-ineffective interventions (based on all studies *n* = 251)

	Unadjusted			Adjusted		
	OR	95% CI	*p*	OR	95%CI	*p*
Category						
Med	Ref					
BI	1.594	0.427 to 5.942	0.488			
Med + BI	5.313	0.588 to 47.976	0.137			
Comp	0.599	0.204 to 1.756	0.35			
Med + Comp	2.266	0.408 to 12.590	0.35			
Mass media	NA	NA	NA			
Control condition						
Nothing/UC	Ref			Ref		
Lower impact	5.332	1.225 to 23.217	.026*	5.374	1.200 to 23.674	.026*
Matched impact	0.699	0.216 to 2.265	0.551	0.733	0.219 to 2.450	0.614
Intervention intensity						
Low	Ref			Ref		
Medium	0.721	0.230 to 2.257	0.574	0.691	0.218 to 2.190	0.53
High	0.28	0.110 to 0.709	.007*	0.283	0.112 to 0.727	.009*
Unclear	NA	NA	NA	NA	NA	NA
Setting						
Primary care	Ref					
Secondary care	0.705	0.146 to 3.400	0.663			
Community	0.38	0.154 to 0.941	.037*			
Workplace	NA	NA	NA			
Unclear/other	0.383	0.125 to 1.171	0.92			
Delivery mode						
Physician	NA	NA				
HP	Ref					
Media	NA	NA				
Mix	NA	NA				
Unclear/other±	2.502	0.721 to 8.688	0.149			
Target level						
Individual	Ref					
Groups	1.514	0.355 to 6.844	0.59			
Population	NA	NA	NA			
Mix	NA	NA	NA			
Population						
Vulnerable	Ref					
General	1.126	0.531 to 2.390	0.756			
Supporting material						
None	Ref					
Self-help	1.49	0.327 to 6.7798	0.606			
Electronic	1.088	0.302 to 3.927	0.897			
Mix	0.745	0.234 to 2.374	0.619			
Pharmacological support	1.488	0.680 to 3.252	0.32			
Social marketing	NA	NA	NA			
Incentives	NA	NA	NA			
Intervention functions						
Training	0.73	0.349 to 2.090	0.73			
Education	0.727	0.298 to 1.772	0.483			
Enablement	1.197	0.559 to 2.562	0.643			
Persuasion	1.143	0.467 to 2.798	0.77			
Environmental restructuring	0.763	0.161 to 3.617	0.733			
Incentivization	1.28	0.157 to 10.461	0.818			
Restriction	NA	NA	NA			
Modeling	NA	NA	NA			
Coercion	NA	NA	NA			
BCT groupings						
Shaping knowledge	0.189	0.025 to 1.433	0.107			
Antecedents	1.431	0.672 to 3.045	0.353			
Regulation	1.499	0.694 to 3.237	0.302			
Social support	0.626	0.294 to 1.333	0.224			
Comparison of outcomes	0.68	0.318 to 1.453	0.32			
Feedback and monitoring	1.078	0.457 to 2.541	0.863			
Goals and planning	0.734	0.326 to 1.653	0.456			
Natural consequences	0.481	0.215 to 1.074	0.074			
Self-beliefs	0.982	0.400 to 2.416	0.969			
Repetition and substitution	0.52	0.180 to 1.504	0.227			
Comparison of behavior	0.68	0.318 to 1.453	0.32			
Associations	0.933	0.260 to 3.344	0.916			
Reward and threat	0.634	0.172 to 2.346	0.495			
Identity	NA	NA	NA			
Scheduled consequences	NA	NA	NA			
Covert learning	NA	NA	NA			
Number of BCTS	0.931	0.836 to 1.036	0.189			

Note: *Significance, all studies were included in the binary analysis of cost-effective versus cost-ineffective interventions (*n* = 251).

NA, not applicable as complete separation.

## Discussion

Around a third of cost-effective interventions were of low intensity, mostly set in primary care and delivered by healthcare professionals. Although there was a large amount of variation across the six behavioral domains, increasing knowledge and/or understanding through education and imparting skills through training were the most prevalent intervention functions, while few used restriction, modeling or coercion. The majority of interventions included around 5% of the potential BCTs specified in the 93-item taxonomy, with the most prevalent BCT groupings being shaping knowledge, antecedents, regulation and social support. Several intervention features were associated with greater cost-effectiveness (those targeting groups or a mixture of groups and individuals versus individuals only; those aimed at the general versus vulnerable populations; and those based on social-marketing) and lower cost-effectiveness (matched control group versus usual care; high intensity versus low intensity; and those offering self-help materials). In terms of intervention functions, those based on training, persuasion and restriction yielded better cost-effectiveness estimates and those based on environmental restructuring and incentivization worse estimates. Several BCT groupings were also associated with greater cost-effectiveness (goals and planning and comparison of behavior) or lower cost-effectiveness estimates (natural consequences and reward and threat).

Extensive evidence exists for the effectiveness of the most prevalent intervention functions and BCT clusters. For example, educational materials and imparting knowledge have been shown to increase the uptake of cervical cancer screening [23], social support appears beneficial in weight loss maintenance [24], restructuring the environment (e.g., removing alcohol) and avoiding exposure to alcohol related cues reduces alcohol consumption [25], and coping skills training in relapse prevention can help those with dependency disorders [26, 27]

At the same time, several functions and BCTs which have demonstrated efficacy were underused or neglected. For example, few used the principles of operant learning (e.g., techniques which involve the manipulation of environmental contingencies such as rewarding behavior, using prompts and cues, agreeing on a behavioral contract and encouraging practice) or encouraged the construction of a new self-identity, both of which underpin much of human behavior [28–31]. This could be because of insufficient intervention descriptions in published/available information [32] or it may reflect intervention developers’ narrow implicit theoretical assumptions regarding causes of behavior and how it might be changed.

It is perhaps unsurprising that interventions were deemed less cost-effective if study design (e.g., matched impact control versus usual care), mode of delivery (e.g., to the individual rather than group) and the intervention itself (e.g., intensity and the provision of self-help) were more complex. This should nonetheless be considered during intervention design. The fact that the incremental effect of the intervention was smaller in more closely matched impact controls, underlines the need to examine mechanisms and intervention processes through the use of appropriate control groups. Choice of control groups or comparison strategies is acknowledged to influence ICER [33]. Usual care is commonly recommended for pragmatic trials which aim to improve current practice and is the primary recommended control group for the calculation of ICERs by NICE [34]. Usual care reflects the care usually received by patients in daily practice and therefore the current “gold standard” [35, 36]. However, usual care controls have also been criticized. Usual care may include many sources of variance and therefore results may not generalize and larger sample sizes may be required. Although conversely, they more likely reflect real-world practice and therefore enhance ecological validity [37].

Use of vulnerable populations also likely increase cost due to difficulties in recruitment and the additional care needed during the programmes’ implementation. Interventions coded for social marketing were deemed particularly cost-effective. Social marketing has been defined as the application of concepts and techniques drawn from the commercial sector (e.g., the four P’s of marketing: Product, Price, Place and Promotion) to promote changes in socially important health behaviors such as drug use and smoking [38]. Previous studies have found that social marketing can form an effective framework for behavior change interventions and provide a useful toolkit for organizations that are trying to change health behaviors [39, 40]. However, findings highlight an ongoing lack of use or underreporting of the use of theory in social marketing campaigns and this may limit its effectiveness [39].

Although the available evidence suggests that financial incentive interventions are more effective than usual care for encouraging healthy behavior change, in our analysis rewards were associated with lower cost-effectiveness [41]. This may be because rewards do not necessarily match up to financial incentives and few studies were explicitly coded as providing incentives and so power may have been low to detect an effect. Environmental restructuring (i.e. removing or adding objects to the environment) and natural consequences, which involves providing information on social, health and emotional consequences, monitoring of emotional consequences and inducing regret, were also associated with lower cost-effectiveness. This would support arguments against a focus on approaches such as that advocated by “Nudge,” which is based on changing the surrounding environment, some incentivization and forms of subtle persuasion to influence behavior, eschewing the use of coercion or other BCW intervention functions [10, 12, 42].

In contrast, goals and planning and comparison of behavior were associated with greater cost-effectiveness. Forming detailed plans of what, when, and how to achieve behavior change have been found to be effective across behavioral domains [43]. Implementation intentions, which take the format of if–then plans, have also been found to be effective not only in promoting initial changes in behavior [44], but also enduring long term changes [45]. Demonstration of behavior and social comparison form part of several behavior change theories including Social Comparison Theory and Social Learning Theory [46, 47], and have been associated previously with smoking cessation success [48], perception of alcohol-related negative consequences [49] and greater weight loss [50].

### Implications

These findings have several implications. First, they may aid evidence-based practice and the application of BCIs in the public domain by providing some of the key BCTs associated with cost-effectiveness. Secondly, studying the types of components of behavioral interventions in this manner may help enable scientific replication, by clearly specifying which components have been employed previously [51]. Finally, elucidating and summarizing the components of interventions may be a valuable resource to intervention designers, with guidelines recommending a full literature review of the components of efficacious interventions before development [52]. As further data accumulates, it will be important to assess if these findings are applicable to individual behavioral domains and other health behaviors not considered in the current review.

### Limitations

To our knowledge, this is the first attempt to synthesize BCIs in terms of their functions and “active ingredients” and to assess the association with cost-effectiveness. However, this study also has several limitations. First, the BCT taxonomy coding approach was applied conservatively, in that a technique was coded as present only when there was unequivocal evidence from written materials that it was used. This is problematic since many intervention reports are poorly specified [53]. Secondly, it is not possible to make a causal attribution of cost-effectiveness to specific BCTs because the BCIs typically contain many of these. Although multiple regression can be used to help discern these effects, caution should be taken during interpretation due to the small sample sizes and possible lack of power i.e. a non-significant effect may reflect no effect or data insensitivity. Thirdly, this paper used the NICE threshold of cost effectiveness of £20,000−£30,000 per QALY. However, there is debate about the correct level of this threshold which is considered implicit rather than explicit [54] and varies enormously between countries [55]. Fourthly, irrespective of the methodology used to evaluate cost-effectiveness, relatively few interventions were considered not to be cost-effective, which likely reduced our ability to detect anything other than relatively large associations with interventions being cost-effective or not. Finally, due to the number of papers (particularly for alcohol and physical activity interventions) it was not possible to consider the predictors of cost-effectiveness as a function of behavioral domain. It remains possible that a combination of functions and techniques are more effective for a given health behavior and that the results do not generalize to health behaviors not included in the current review.

## Conclusion

In conclusion, this study reliably categorized and coded the BCTs used in BCIs across six behavioral domains and assessed the association with cost-effectiveness. These interventions heavily relied on education and training, with substantial variations found across the interventions targeting the six health behaviors. Although most interventions used relatively few BCTs, those employing goal setting and comparison of behavior were deemed more cost-effective. These findings will be of interest to intervention developers and policy makers attempting to implement BCIs in the real world.

## Supplementary Material

kaab036_suppl_Supplementary_File
