# Supplementary material for: Behavioral Intervention Components Associated With Cost-effectiveness: A Comparison of Six Domains
Source: Ann Behav Med. 2021 Jun 11;56(2):176–92. doi: 10.1093/abm/kaab036 (PMC8832109; doi:10.1093/abm/kaab036)
Supplement: kaab036_suppl_Supplementary_File [file kaab036_suppl_supplementary_file.docx]

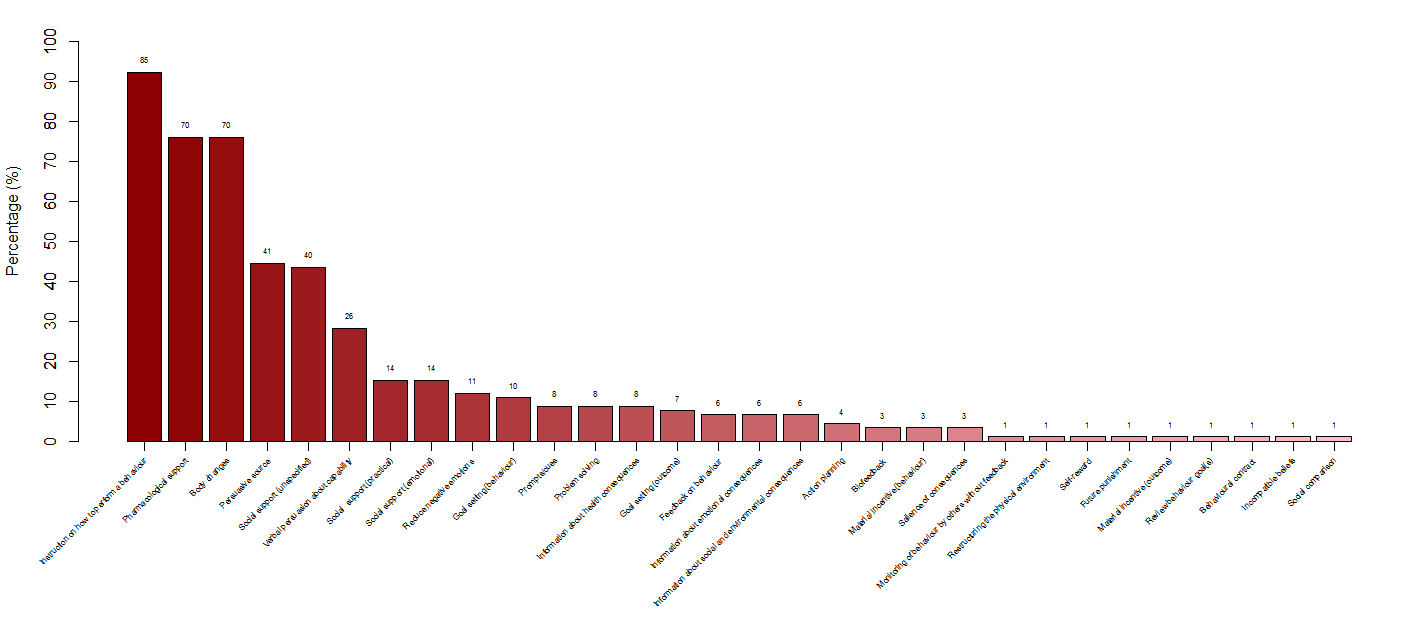


**Supplementary Figure 1:** Prevalence of individual BCTs across smoking cessation interventions

*Note: Only BCTs described in at least one interventions are shown*


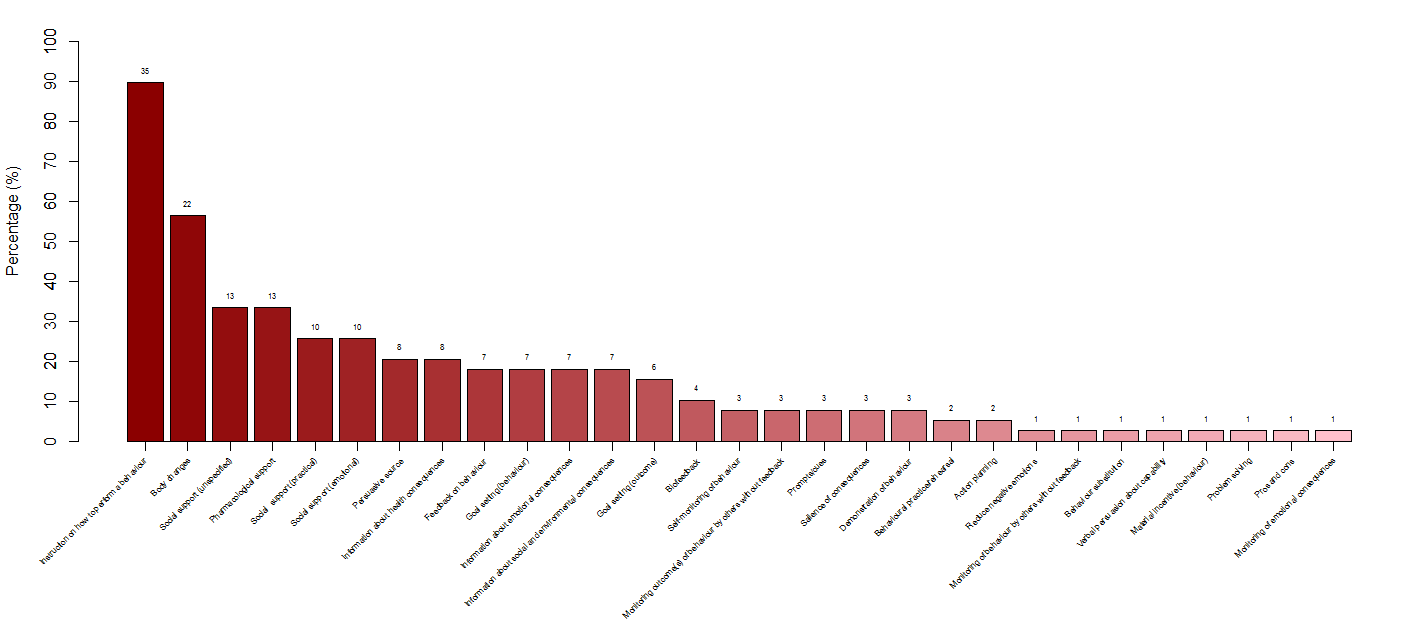


**Supplementary Figure 2:** Prevalence of individual BCTs across diet interventions

*Note: Only BCTs described in at least one interventions are shown*


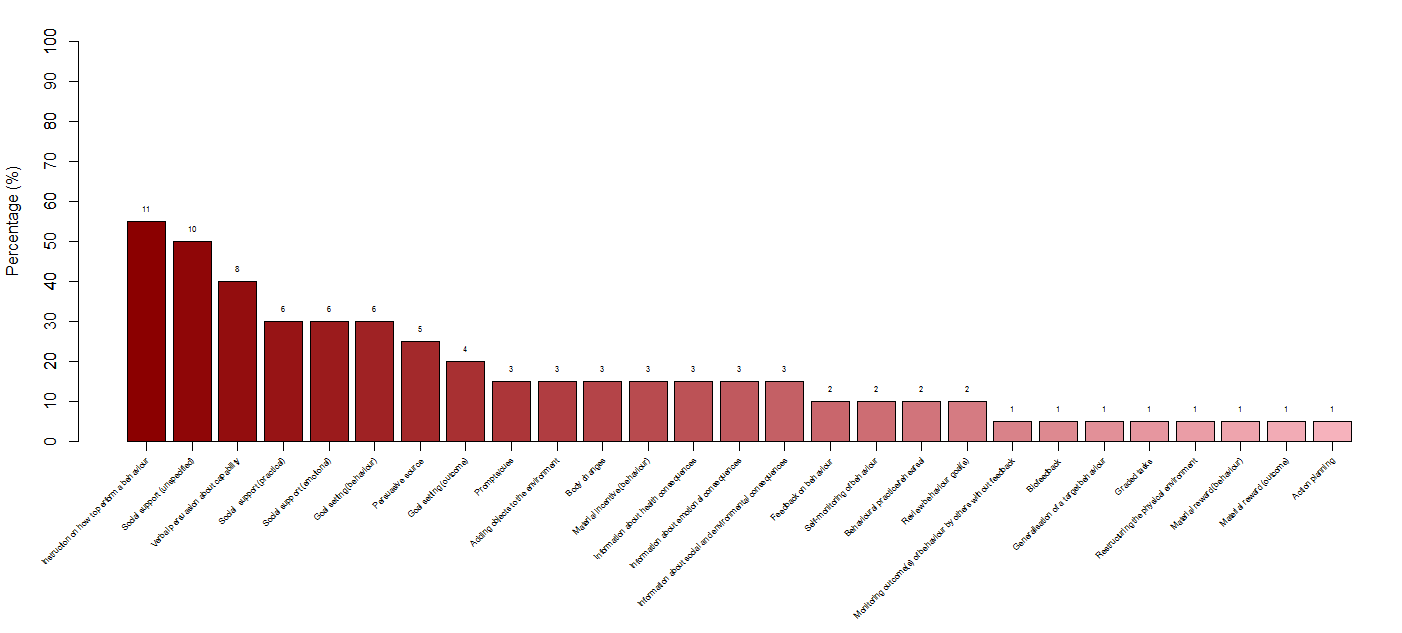


**Supplementary Figure 3:** Prevalence of individual BCTs across physical activity interventions

*Note: Only BCTs described in at least one interventions are shown*


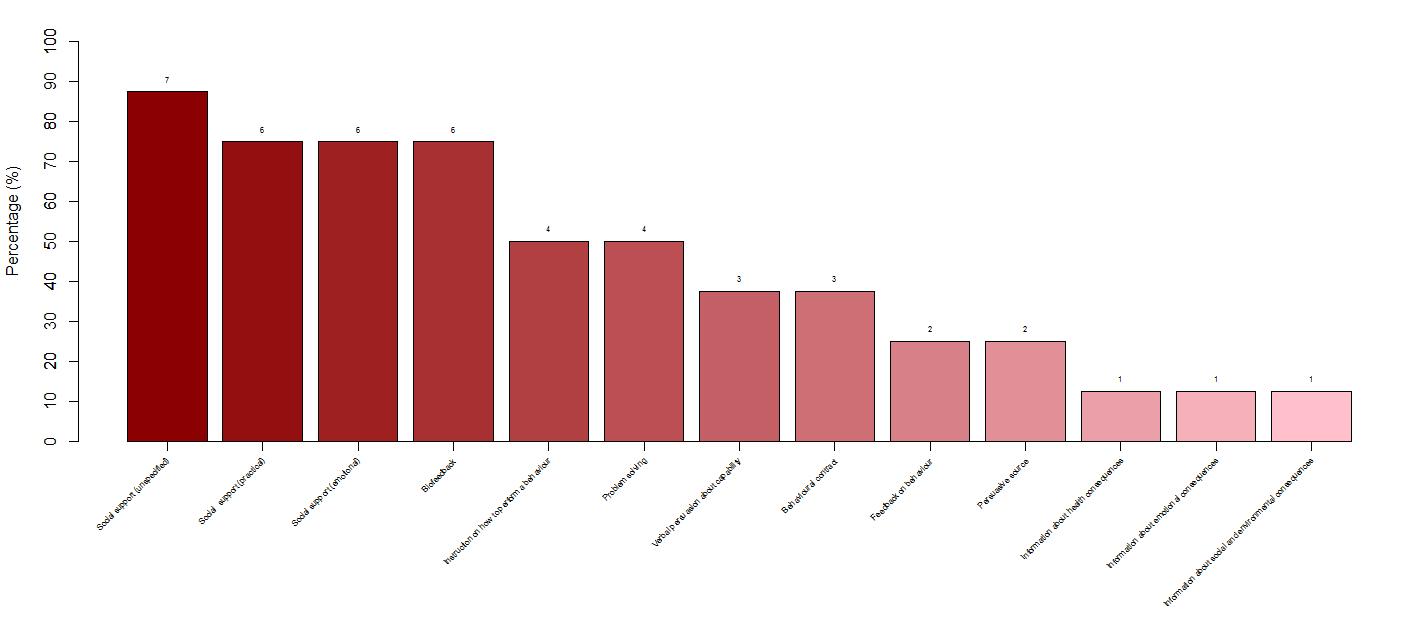


**Supplementary Figure 4:** Prevalence of individual BCTs across alcohol interventions

*Note: Only BCTs described in at least one interventions are shown*


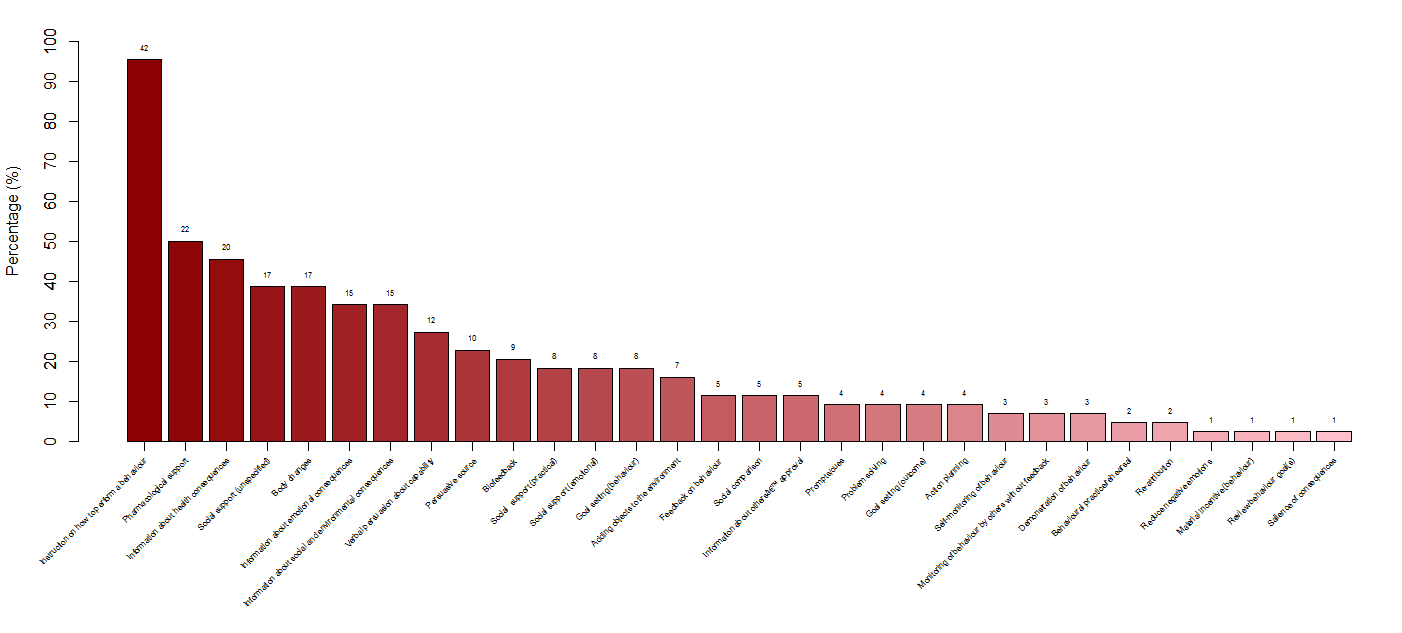


**Supplementary Figure 5:** Prevalence of individual BCTs across sexual health interventions

*Note: Only BCTs described in at least one interventions are shown*


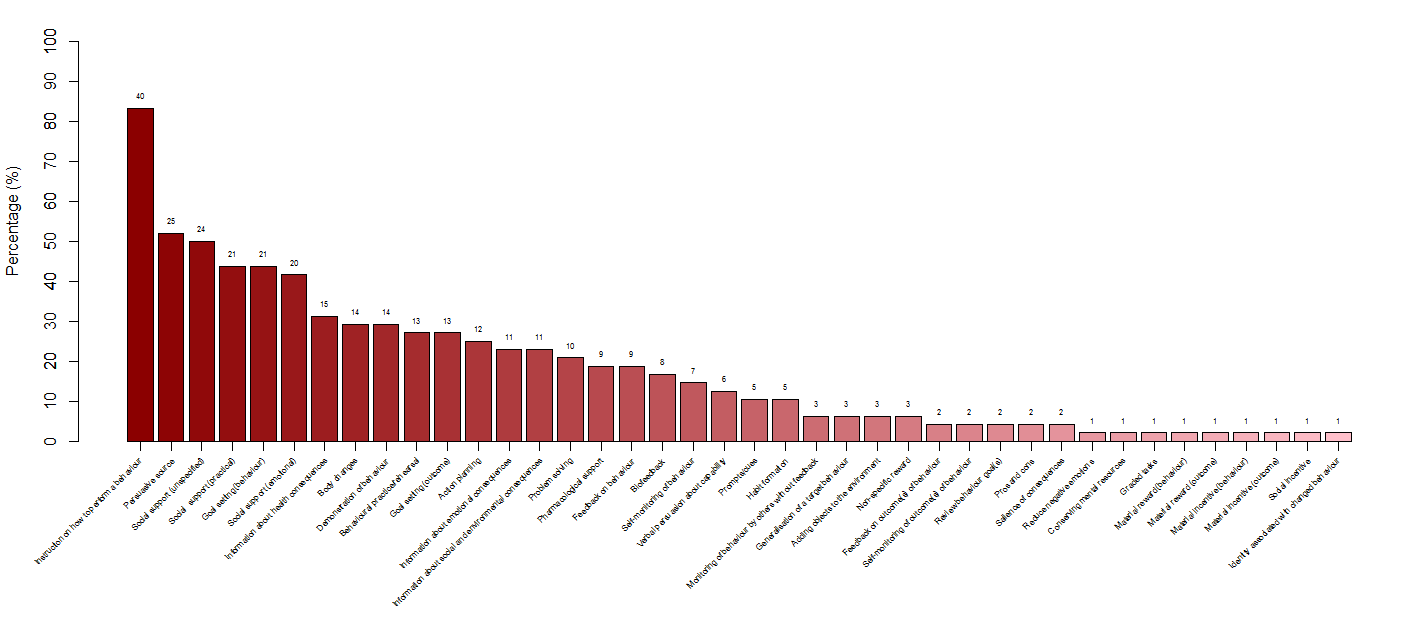


**Supplementary Figure 6:** Prevalence of individual BCTs across multiple health behaviour interventions

*Note: Only BCTs described in at least one interventions are shown*

**Supplementary Table 1:** Intervention functions

| ***Intervention type*** | ***Definition*** | **Examples** |
| --- | --- | --- |
| Education | Increasing knowledge or understanding | Providing information to promote healthy eating |
| Persuasion | Using communication to induce positive or negative feelings or stimulate action | Using imagery to motivate increases in physical activity |
| Incentivisation | Creating expectation of reward | Using prize draws to induce attempts to stop smoking |
| Coercion | Creating expectation of punishment or cost | Raising the financial cost to reduce excessive alcohol consumption |
| Training | Imparting skills | Advanced driver training to increase safe driving |
| Restriction | Using rules to reduce the opportunity to engage in the target behaviour (or to increase the target behaviour by reducing the opportunity to engage in competing behaviours) | Prohibiting sales of solvents to people under 18 to reduce use for intoxication |
| Environmental restructuring | Changing the physical or social context | Providing on-screen prompts for GPs to ask about smoking behaviour |
| Modelling | Providing an example for people to aspire to or imitate | Using TV drama scenes involving safe-sex practices to increase condom use |
| Enablement | Increasing means/reducing barriers to increase capability or opportunity^1^ | Behavioural support for smoking cessation, medication for cognitive deficits, surgery to reduce obesity, prostheses to promote physical activity |

Note: Adapted from [^10^](#_ENREF_10)

**Supplementary Table 2:** Broad categorisation of interventions

| Category distinctions | Description |
| --- | --- |
| Intervention intensity | Low: one face-to-face contact or other direct contact lasting up to 5 minutes or any non-specific (impersonal, e.g. through media) contact  Medium: one face-to-face contact or other direct contact lasting more than 5 minutes, or one face-to-face contact or other direct contact lasting up to 5 minutes on more than one occasion  High: any face-to-face contact or other direct contact lasting more than 5 minutes on more than one occasion. |
| Setting | Primary or secondary care  Community  Workplace  Other |
| Mode of delivery | Physician  Health-care professional (included nurses, pharmacists, psychologists, dieticians and other qualified personnel)  Media  Combination  Other/not specified |
| Target level | Individual  Group  Population |
| Supporting material | Self-help (written)  Electronic (e.g. telephone, mobile phone, computer)  Mixture  None |
| Use of pharmacological support | It was recorded whether or not pharmacological support was provided in addition to the type of medication (e.g. NRT for smoking cessation) |
| Use of incentives | It was recorded whether or not incentives were provided to participate in the study and continue with the intervention |
| Social marketing | It was recorded whether social-marketing was implemented during the intervention. Social Marketing was defined as the process of using principles and techniques to influence target audience behaviors that will benefit society. |
| Population | General population  Vulnerable population (e.g. pregnant women, individuals at risk of disease, and those from lower socio-economic groups) |
